# Rapid Point-of-Care Genotyping to Avoid Aminoglycoside-Induced Ototoxicity in Neonatal Intensive Care

**DOI:** 10.1001/jamapediatrics.2022.0187

**Published:** 2022-03-21

**Authors:** John H. McDermott, Ajit Mahaveer, Rachel A. James, Nicola Booth, Mark Turner, Karen E. Harvey, Gino Miele, Glenda M. Beaman, Duncan C. Stoddard, Karen Tricker, Rachel J. Corry, Julia Garlick, Shaun Ainsworth, Thomas Beevers, Iain A. Bruce, Richard Body, Fiona Ulph, Rhona MacLeod, Peter L. Roberts, Paul M. Wilson, William G. Newman

**Affiliations:** 1 Manchester Centre for Genomic Medicine, St Mary’s Hospital, Manchester University NHS Foundation Trust, Manchester, United Kingdom; 2 School of Biological Sciences, Division of Evolution, Infection and Genomics, University of Manchester, Manchester, United Kingdom; 3 Newborn Intensive Care Unit, Manchester University NHS Foundation Trust, Manchester, United Kingdom; 4 Newborn Intensive Care Unit, Liverpool Women’s Hospital, Liverpool, United Kingdom; 5 Genedrive Diagnostics Ltd, Manchester, United Kingdom; 6 DS Analytics and Machine Learning Ltd, Hammersmith, London, United Kingdom; 7 Hearing Health Theme Manchester NIHR Biomedical Research Centre, Manchester, United Kingdom; 8 Pediatric ENT Department, Royal Manchester Children’s Hospital, Manchester University NHS Foundation Trust, Manchester, United Kingdom; 9 Emergency Department, Manchester University NHS Foundation Trust, Manchester, United Kingdom; 10 Division of Cardiovascular Sciences, University of Manchester, Manchester, United Kingdom; 11 Manchester Centre for Health Psychology, Division of Psychology & Mental Health, School of Health Sciences, Faculty of Biology, Medicine and Health, University of Manchester, Manchester Academic Health Science Centre, Manchester, United Kingdom; 12 Market Access & Reimbursement Solutions Ltd, Liverpool, Merseyside, United Kingdom; 13 Alliance Manchester Business School, University of Manchester, Manchester, United Kingdom

## Abstract

**Importance:**

Aminoglycosides are commonly prescribed antibiotics used for the treatment of neonatal sepsis. The *MT-RNR1* m.1555A>G variant predisposes to profound aminoglycoside-induced ototoxicity (AIO). Current genotyping approaches take several days, which is unfeasible in acute settings.

**Objective:**

To develop a rapid point-of-care test (POCT) for the m.1555A>G variant before implementation of this technology in the acute neonatal setting to guide antibiotic prescribing and avoid AIO.

**Design, Setting, and Participants:**

This pragmatic prospective implementation trial recruited neonates admitted to 2 large neonatal intensive care units between January 6, 2020, and November 30, 2020, in the UK.

**Interventions:**

Neonates were tested for the m.1555A>G variant via the rapid POCT on admission to the neonatal intensive care unit.

**Main Outcomes and Measures:**

The primary outcome assessed the proportion of neonates successfully tested for the variant of all infants prescribed antibiotics. Secondary outcomes measured whether implementation was negatively associated with routine clinical practice and the performance of the system. The study was statistically powered to detect a significant difference between time to antibiotic administration before and after implementation of the *MT-RNR1* POCT.

**Results:**

A total of 751 neonates were recruited and had a median (range) age of 2.5 (0-198) days. The *MT-RNR1* POCT was able to genotype the m.1555A>G variant in 26 minutes. Preclinical validation demonstrated a 100% sensitivity (95% CI, 93.9%-100.0%) and specificity (95% CI, 98.5%-100.0%). Three participants with the m.1555A>G variant were identified, all of whom avoided aminoglycoside antibiotics. Overall, 424 infants (80.6%) receiving antibiotics were successfully tested for the variant, and the mean time to antibiotics was equivalent to previous practice.

**Conclusions and Relevance:**

The *MT-RNR1* POCT was integrated without disrupting normal clinical practice, and genotype was used to guide antibiotic prescription and avoid AIO. This approach identified the m.1555A>G variant in a practice-changing time frame, and wide adoption could significantly reduce the burden of AIO.

## Key Points

**Question:**

Can rapid point-of-care genotyping technology be implemented in the acute neonatal setting to avoid aminoglycoside-induced ototoxicity without disrupting normal standards of care?

**Findings:**

In this pragmatic prospective implementation trial that included 751 neonates in the UK, a 26-minute rapid genotyping platform was successfully implemented, identified neonates at risk of aminoglycoside-induced ototoxicity, facilitated tailored prescribing, and did not disrupt normal clinical practice.

**Meaning:**

Rapid genetic point-of-care testing may be used to avoid aminoglycoside-induced ototoxicity in neonates, and clinicians are able to integrate genetic data into their routine practice in the acute setting.

## Teaser

This pragmatic prospective implementation trial uses a rapid genotyping platform for the m.1555A>G variant to assess whether this technology could be implemented to avoid aminoglycoside-induced ototoxicity without disrupting normal clinical practice on neonatal intensive care units.

## Introduction

Aminoglycoside antibiotics are commonly used worldwide to treat gram-negative infections. They have proven efficacy and their safety profile is well recognized.^1^ In high doses, or with protracted regimens, aminoglycosides cause nephrotoxicity and ototoxicity, the latter of which can manifest as either vestibulotoxicity or cochleotoxicity.^2^ It was noted in the latter part of the 20th century that certain families have a predisposition to aminoglycoside-induced ototoxicity (AIO), whereby a single dose could cause profound and irreversible hearing loss.^3,4^

Consistent with matrilineal inheritance in these families, in 1993, this adverse drug reaction was shown to be due to a mitochondrial genome variant in *MT-RNR1*, m.1555A>G.^5^ The m.1555A>G variant causes a conformational change in the structure of the eukaryotic 12S ribosomal RNA subunit, meaning that it more closely resembles the bacterial 16S ribosomal RNA subunit, allowing aminoglycosides to bind. The impact of this increased affinity is most notable within the inner ear hair cells, leading to cellular damage and AIO.^6,7,8^

Guidance from the Clinical Pharmacogenetics Implementation Consortium and from the UK Medicines and Healthcare products Regulatory Agency have recently been published on prescription of aminoglycoside antibiotics in individuals with the m.1555A>G variant.^9,10^ Despite uncertainty around the penetrance of the variant, the risk has been deemed sufficiently high that recommendations were made that individuals with the m.1555A>G variant should not receive aminoglycoside antibiotics unless the high risk of permanent hearing loss is outweighed by the severity of infection and lack of safe or effective alternative therapies.^9,10^

Intravenous benzylpenicillin and the aminoglycoside antibiotic, gentamicin, are routinely prescribed in combination as the first-choice regimen for empirical treatment of infection in the neonatal period.^11^ In the context of neonatal infection, efficacious alternatives exist, such as cephalosporins, but their broad-spectrum activity promotes the development of antibiotic resistant organisms. As such, aminoglycosides remain the preferred first-line agent, but for individuals with the m.1555A>G variant, a cephalosporin-based regimen would be appropriate.

As antibiotics should be delivered within an hour of any decision to treat sepsis, current genetic technologies are not sufficiently rapid to genotype m.1555A>G within a clinically relevant time frame in the acute setting.^12^ We developed a rapid genotyping platform for the m.1555A>G variant and assessed whether this technology could be implemented to avoid AIO without disrupting normal clinical practice on neonatal intensive care units (NICUs) via a prospective implementation trial.

## Methods

The study was approved by the National Health Service Research Ethics Committee (IRAS 253102) and the Human Research Authority. Requirement for prospective consent was waived by the National Health Service Research Ethics Committee and Human Research Authority. *MT-RNR1* testing was considered an excepted purpose under the UK’s Human Tissue Act 2004, and therefore DNA analysis without prospective consent was permissible. This novel ethical framework, necessitated by the acute context in which testing is being undertaken, has been discussed elsewhere extensively.^13,14,15^

Given the relatively low prevalence of the m.1555A>G variant in the United Kingdom, approximately 0.2%, an unfeasibly large sample size would be required to estimate the sensitivity of the point-of-care test (POCT) using a traditional prospective diagnostic test accuracy study.^16^ Therefore, we evaluated the test in 2 stages. First, the assay underwent preclinical validation via a case-control study followed by a prospective trial to evaluate the real-world impact of implementing the POCT in the neonatal setting.

### *MT-RNR1* Assay Development and Validation

The Genedrive platform is a rapid thermocycling POCT instrument that was programmed to perform loop-mediated isothermal amplification followed by fluorescent hybridization probe-based melt analysis to enable allelic discrimination. The assay was designed to genotype m.1555A>G from a buccal swab to differentiate wild-type and variant alleles by melt peak analysis (eFigure 1 in Supplement 1).

Preclinical analytical specificity was assessed using a case-control clinical performance study with buccal samples from neonates, infants, and adults. The assay was tested on samples from individuals of different ethnic groups, assessed for cross reactivity, and the effect of potential interfering substances was measured. Further technical specifications are provided in the eAppendix in Supplement 1.

### Trial Oversight

The Pharmacogenetics to Avoid the Loss of Hearing (PALOH) trial was an investigator-initiated, pragmatic prospective implementation trial (ISRCTN13704894).^17^ The trial was undertaken at 2 large NICUs in the United Kingdom. Both centers comply with UK National Institute for Clinical Excellence (NICE) guidelines for the treatment of neonatal infection.^11^ This guidance states that the first-line antibiotic regimen for infants up to and including 28 days of age with suspected bacterial infection should be benzylpenicillin plus gentamicin. At the study centers, infants older than 28 days were prescribed cefotaxime plus gentamicin (study site 1) or co-amoxiclav plus gentamicin (study site 2) based on local antimicrobial guidelines. As such, all infants with suspected bacterial infection on the NICUs were considered for an aminoglycoside-based regimen. A trial management committee (eTable 4 in Supplement 1) and an independent steering group (eTable 5 in Supplement 1) were established to provide oversight for the project. 

### Participants

All neonates admitted to NICUs across the 2 participating sites were eligible for enrollment. Neonates were recruited from January 6, 2020, to November 30, 2020, and enrolled on admission and included those admitted directly from the delivery suite, transferred from other wards, or from an external neonatal unit. Neonates requiring antibiotics immediately, as determined by the admitting clinician, with established intravenous access, were excluded. A complete list of the inclusion and exclusion criteria for each recruiting site is provided in the eAppendix in Supplement 1 and has been published.^17^ Data on race and ethnicity were not collected.

### Trial Procedures

During the study period, the m.1555A>G buccal swab was performed on admission. These were undertaken by the admitting nurse at the same time as skin swabs, which are standardly performed at both centers, as part of routine clinical practice, to screen for methicillin-resistant *Staphylococcus aureus*.^18^ Once the *MT-RNR1* result was available, it was used to guide antibiotic prescribing, avoiding aminoglycoside antibiotics and prescribing an alternative regimen, consistent with existing national guidelines, if the m.1555A>G variant was detected.^19^ Neonates with the m.1555A>G variant were referred for family counseling to the regional clinical genetics service. Study data were recorded by the admitting nurse and a study administrator.

### Outcomes

The primary outcome was the number of neonates successfully tested for the m.1555A>G variant as a proportion of all infants who received aminoglycoside antibiotics. The secondary outcomes were the total number of neonates identified with the m.1555A>G genetic variant and whether they avoided aminoglycosides; the proportion of neonates where testing was not undertaken; the median time to swab; and the mean time to antibiotic administration. The mean time to antibiotic administration was compared against data measuring clinical timings of admissions to NICU over a month-long period prior to study initiation. All buccal samples were genotyped at the conclusion of the study to determine the real-world analytical performance of the *MT-RNR1* system. The accuracy of the Genedrive *MT-RNR1*-ID kit was compared against Sanger sequencing.

### Statistical Analysis

Given the low frequency of the variant, the study was not explicitly powered to ensure detection of a m.1555A>G variant. Rather, the study was designed to detect a significant difference between time to antibiotic administration before and after implementation of the *MT-RNR1* POCT. To achieve a statistical power of 90% for a null hypothesis statistical test, with a 5% significance level, a Cohen *d* margin of 0.5 and an assumed control sample of 95, at least 54 recruits would be required, within the 10-month recruitment estimate.

Analysis of the primary outcome was performed via descriptive statistics. For all admissions, a standardized time zero was calculated, against which clinical timings could be measured. Where antibiotics were prescribed, the time that a decision was made to prescribe antibiotics represents time zero. If antibiotics were not prescribed, then time zero was represented by the time of admission onto NICU.

Differences between time to antibiotic administration before and during the study were assessed using a null hypothesis statistical test for a difference in mean times and a two one-sided *t* test to test for equivalence. The quantity of interest was the mean difference between time zero and the time of antibiotic administration before (μ_c_) and during the study (μ_t_) for those admissions prescribed antibiotics within the first 2 hours of admission. The null hypothesis for the null hypothesis statistical test is that the difference in mean times is zero, ie, H_0_μ_c_ – μ_t_ = 0. The null hypothesis for the two one-sided *t* test is that the difference between these quantities is greater than or equal to a Cohen *d* (0.5) margin (Δ), ie, H_01:_μ_c_ – μ_t_≤– Δ; H_02 _: μ_c _– μ_t≥_Δ.

### Patient and Public Involvement

Involvement of parent and public representatives has been a critical component of the development of this trial protocol. Parent representatives are involved in both the trial management groups, stakeholders committee (eTable 6 in Supplement 1) and a separate Public and Patient Involvement and Engagement panel, providing both neonatal care and/or hearing loss experience. The Public and Patient Involvement and Engagement panel was involved in the development of both the protocol and ethics application. The principal investigator and coinvestigators presented early versions of the texts to the Public and Patient Involvement and Engagement panel and any potentially contentious issues were discussed in more detail. The insights gained from these meetings were used to refine the applications prior to submission.

## Results

### Preclinical Assay Validation

Buccal samples were collected and genotyped (n = 304) for preclinical assay validation from 159 individuals (eTable 1 in Supplement 1). Preclinical validation demonstrated an assay sensitivity of 100% (95% CI, 93.9%-100.0%), a specificity of 100% (95% CI, 98.5%-100.0%), and time to genotype of 26 minutes. The limit of detection, defined as the lowest concentration at which more than 95% of the tested samples generated a positive result, was 16 cells. The *MT-RNR1* assay was inclusive across all ethnicities tested, possible interfering substances did not impact performance, and there was no cross-reactivity (eAppendix, eTable 2, and eTable 3 in Supplement 1). Further technical specifications are available in the eAppendix in Supplement 1.

### Participant Characteristics

**Figure 1.**
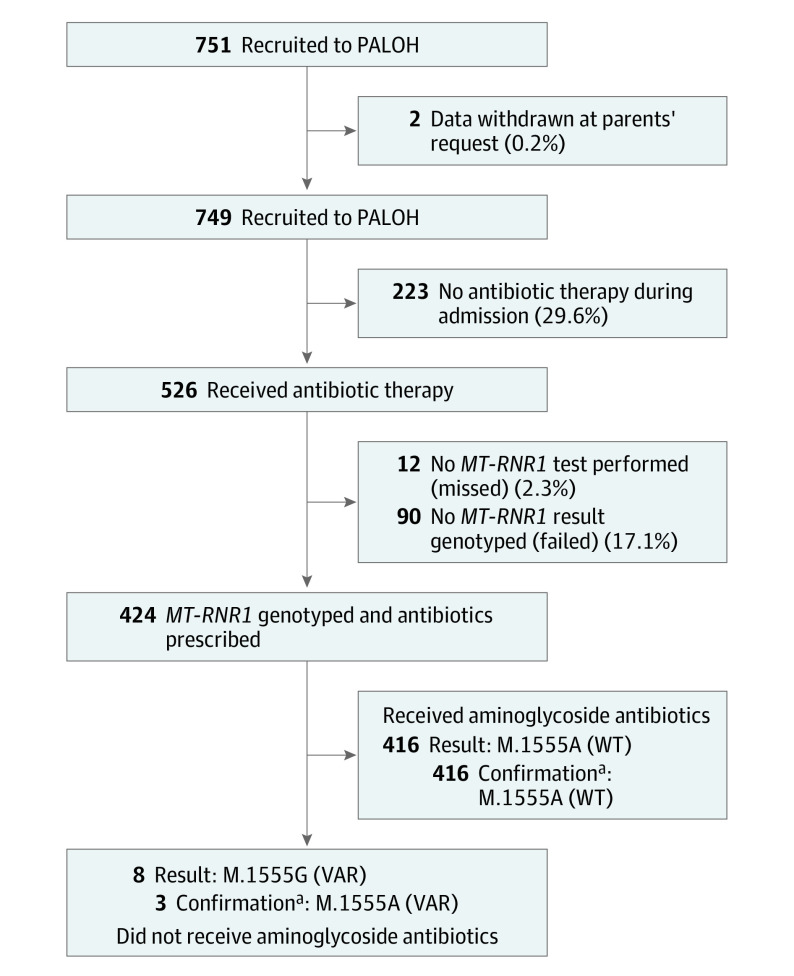
Study Recruitment and Testing Metrics Flow diagram demonstrating recruitment to the Pharmacogenetics to Avoid the Loss of Hearing (PALOH) trial, antibiotic therapy, and the outcome of *MT-RNR1* testing. VAR indicates variant; WT, wild type. ^a^Confirmation of m.1555 genotype via sanger sequencing.

Overall, 751 neonates were recruited from 2 centers (Figure 1). Most neonates (713 [94.9%]), were recruited from a single center because recruitment was paused at the second center and did not recommence owing to the SARS-CoV-2 outbreak. The steering committee agreed that recruitment would be extended for an additional month to compensate for the withdrawn center. Participants’ median (range) age was 2.5 (0-198) days at the time of recruitment. Mean (SD) gestational age at time of delivery was 37 (4) weeks. A total of 526 neonates (70%) received antibiotics as part of their care. Two neonates had their data withdrawn from the study at the request of their parents.

### *MT-RNR1* Assay Performance

Overall, 424 neonates (80.6%) prescribed aminoglycoside antibiotics were successfully tested for the m.1555A>G variant. Three neonates with the m.1555A>G variant were identified and confirmed by Sanger sequencing. Of the 526 admissions who received antibiotics, 102 (19.4%) were not successfully tested; 90 tests (17.1%) failed and 12 eligible patients (2.3%) were not tested by the clinical teams (Figure 1). Of the whole cohort, 153 admissions (20.4%) were not successfully tested; 128 tests (17.1%) failed and 25 admissions (3.3%) were not tested by the clinical teams. There were 5 false-positive results. There were no false-negative results. As such, the assay had a real-world analytical sensitivity of 100% (95% CI, 29.2%-100.00%), a specificity of 99.2% (95% CI, 98.0%-99.7%), and an accuracy of 99.2% (95% CI, 98.0%-99.7%).

Throughout the trial, the *MT-RNR1* platform was updated to improve efficiency (proportion of tests returning valid results) and accuracy. This iterative process identified a cause of the false-positive results as incomplete cartridge insertion, permitting light ingress past the reaction cartridge during the melting phase, a defect that was eliminated with an update to the cartridge design to ensure complete cartridge assembly and insertion. Unsuccessful genotyping (test fail) was found to be predominantly associated with low-signal intensity during the melting phase, which was resolved in the postrecruitment period via modifications to the assay buffer (eFigure 2 in Supplement 1). In combination with the revised cartridge consumable, the redesigned assay demonstrated a reduced failure. Repeated testing of samples where genotyping had previously failed demonstrated an improved failure rate of 5.7% when performed in the intended-use clinical setting and 0% when performed in the laboratory. Further details are provided in the eAppendix in Supplement 1.

### Integration of the System Within Clinical Practice

**Figure 2.**
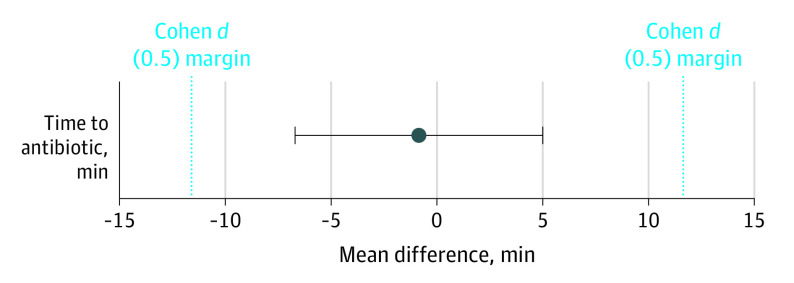
Time to Antibiotic Therapy Comparison of time to antibiotic during reference and study periods. The upper and lower bounds of the confidence interval (95% CI, −5.96 to 4.23 minutes) lie within the Cohen *d* margins. The mean difference in time to antibiotic between reference and study periods is −0.87 minutes.

Prior to implementation, the mean (SD) time to antibiotic therapy was 55.87 (22.56) minutes based on 95 consecutive acute admissions over 1 month. During the study, the corresponding mean (SD) time to antibiotic therapy was 55.18 (23.82) minutes. Before and after implementation of the *MT-RNR1* assay, there was a difference in the mean times to antibiotic therapy of −0.87 minutes (Figure 2).

Both the upper and lower 1-sided *t* tests for equivalence are significant (upper: *P* < .001; lower: *P* < .001) and the bounds of the two one-sided *t* test confidence interval lay within the Cohen *d* margins (95% CI, −5.96 to 4.23 minutes). This suggests statistical equivalence between the mean times to antibiotic in the control and study samples. The null hypothesis statistical test was nonsignificant (97.5% CI, −6.70 to 4.97 minutes; *P* = .74), meaning we failed to reject the null hypothesis that the true difference in mean time to antibiotic is zero. Based on the two one-sided *t* test and the null hypothesis statistical test combined, we concluded that the observed difference in mean time to antibiotic in the 2 samples is statistically equivalent to zero. The median (IQR) time to swab throughout the study was 6 (3-16) minutes. In all cases where a m.1555A>G genotype was identified, aminoglycoside antibiotics were avoided and alternative cephalosporin-based regimens were used (Figure 1).

## Discussion

In this pragmatic implementation trial, clinicians were able to integrate a novel genetic POCT into existing clinical pathways to guide antibiotic therapy and avoid AIO in the acute neonatal setting. Genotyping was successful in the majority of neonates and implementation of the POCT did not negatively impact existing pathways, with time to antibiotic therapy equivalent to previous practice. Only 3.3% of admissions were not tested, demonstrating excellent uptake of the system by clinical teams who had no prior experience of genetic testing or laboratory practice. Critically, where the m.1555A>G genotype was identified, clinicians were able to use that data to practice tailored antibiotic prescribing, avoiding AIO in 3 infants who would have otherwise received gentamicin.

To our knowledge, until this study, there has been no genotyping technology sufficiently rapid to identify the m.1555A>G within a clinically relevant time frame in the context of neonatal sepsis. The UK NICE guidelines for neonatal infection state that if a neonate requires antibiotic therapy, this should be administered within 1 hour of the decision to treat. This is in line with the long-standing concept of the golden hour, a commonly cited concept in the management of sepsis.^20,21,22^ With a run time of 26 minutes, clinical teams were able to perform genotyping within a sufficiently rapid time frame, meaning the mean time to antibiotic therapy was equivalent to previous practice.

There is precedent for the use of POCTs to identify pharmacogenetic variation in clinical practice. Previous studies have reported efforts to rapidly genotype variants related to the metabolism of the anticoagulant warfarin and the antiplatelet clopidogrel.^23,24,25^ Common genetic variation can reduce the efficacy of these medicines and prompt genotyping could provide opportunity to select alternative agents or optimize dosing strategies. Although there are parallels between these studies and the one described here, the testing paradigm for the *MT-RNR1* genotype and aminoglycoside therapy carries an even greater pressure for a rapid and accurate result.

Depending on the clinical context, stakeholders will have their own thresholds for acceptable test performance. In most clinical settings, there is likely to be sufficient time for further genotyping if the initial test fails. This is not the case in neonatal sepsis, where antibiotics are required within the hour and delay could be lethal.^12^ Here, we identified a divergence between laboratory and real-world assay performance, emphasizing the importance of implementation trials in assessing real-world assay performance in the intended use setting. The design of this trial allowed iterative improvements to the system based on study data and clinical feedback, leading to a greatly improved system for implementation. Based on our experience, implementation assessments such as this should form part of any in vitro diagnostic manufacturer’s development pipeline.

The acute context in which this trial is performed creates several novel technical, methodological, and ethical challenges.^13,17^ To our knowledge, this is the first time a genetic POCT has been implemented in the acute neonatal setting to guide treatment. There was a high degree of acceptability for this novel genetic testing approach from clinicians and parents, evidenced by the uptake of testing and by the small number of withdrawals.

Both centers in this study followed the NICE guidelines for treatment of neonatal infection.^11^ When an infant was identified to carry the m.1555A>G variant, cefotaxime monotherapy, a regimen that is considered to have comparable antimicrobial coverage to benzylpenicillin plus gentamicin, was commenced.^26,27^ As such, these individuals received equally efficacious antibiotics but avoided the potential for lifelong hearing loss. Cefotaxime is not recommended as a first-line agent in the United Kingdom for neonatal infection because of its broad spectrum of activity, which could predispose to antimicrobial resistance if widely used.^11^ The integration of the *MT-RNR1* test into routine neonatal practice will likely necessitate the development of local or regional guidelines that reflect local microbial surveillance data and outline appropriate prescribing based on RNR1 genotype.

### Limitations

The PALOH trial recruited patients from 2 large academic teaching hospitals in the UK, both of which have level 4 NICUs, proving the acute care for critically unwell neonates. Owing to the SARS-CoV-2 pandemic, recruitment was mainly from a single site. Both sites engage in research activity on a regular basis and have extensive experience with the implementation of new technologies. This experience facilitated delivery of the PALOH trial but should be considered when attempting to generalize any findings to other centers that may not have equivalent research or implementation expertise. Furthermore, antibiotic prescribing practices show considerable variation, both in the rates of prescription and the agents chosen, both within and between countries. As such, the utility and cost effectiveness of m.1555A>G testing will be context dependent. Local value assessments and implementation programmes should be undertaken as part of a multidisciplinary strategy with stakeholders from NICU, microbiology, pharmacy, and clinical genetics.

## Conclusions

This study demonstrates that genotyping can be performed in the acute setting and incorporated into clinical practice without disrupting existing standards of care. Based on the population frequency of the m.1555A>G variant, and the worldwide use of aminoglycosides in more than 7 million neonates each year, the adoption of a *MT-RNR1* POCT would potentially avoid thousands of AIO cases annually, particularly in low- and middle-income countries where aminoglycosides are widely prescribed.^28^ There are a growing number of acute clinical scenarios where knowledge of an individual’s genotype could be used to improve outcomes, and the SARS-CoV-2 pandemic has led to the proliferation of in vitro diagnostic systems that could be redeployed for rapid genotyping.^29^ This trial demonstrates a technological approach to perform genotyping that allows genotype to be integrated into acute clinical pathways without disrupting normal standards of care.

## Notes

Supplement 1. eFigure 1. Melt Curve Analysis eFigure 2. Peak Height Assessment eAppendix. eTable 1. Interfering substances eTable 2. Cross Reactivity eTable 3. Pre-clinical Assay Validation Case Control Study Participants eTable 4. Trial Management Committee: Monthly Meetings eTable 5. Steering Committee: 6 Monthly Meetings eTable 6. Stakeholders Committee

Supplement 2. Nonauthor Collaborators. The PALOH Study Team members
