# Supplementary material for: Rapid Point-of-Care Genotyping to Avoid Aminoglycoside-Induced Ototoxicity in Neonatal Intensive Care
Source: JAMA Pediatr. 2022 Mar 21;176(5):486–92. doi: 10.1001/jamapediatrics.2022.0187 (PMC8938898; doi:10.1001/jamapediatrics.2022.0187)
Supplement: Supplement 1. — eFigure 1. Melt Curve Analysis eFigure 2. Peak Height Assessment eAppendix. eTable 1. Interfering substances eTable 2. Cross Reactivity eTable 3. Pre-clinical Assay Validation Case Control Study Participants eTable 4. Trial Management Committee: Monthly Meetings eTable 5. Steering Committee: 6 Monthly Meetings eTable 6. Stakeholders Committee [file jamapediatr-e220187-s001.pdf]

Supplemental Online Content

McDermott JH, Mahaveer A, James RA, et al; PALOH Study Team. Rapid point-of-care genotyping to avoid aminoglycoside-induced ototoxicity in neonatal intensive care. *JAMA Pediatr*. Published online March 21, 2022. doi:10.1001/jamapediatrics.2022.0187

eFigure 1. Melt Curve Analysis

eFigure 2. Peak Height Assessment

eAppendix.

eTable 1. Interfering substances

eTable 2. Cross Reactivity

eTable 3. Pre-clinical Assay Validation Case Control Study Participants

eTable 4. Trial Management Committee: Monthly Meetings

eTable 5. Steering Committee: 6 Monthly Meetings

eTable 6. Stakeholders Committee

This supplemental material has been provided by the authors to give readers additional information about their work.

© 2022 McDermott JH et al. *JAMA Pediatrics.*

eAppendix.

1. Assay Background and Development

1.1. Background

The Genedrive® MT-RNR1 ID Kit was developed for use as *In vitro* diagnostic (IVD) molecular assay for the detection of the single nucleotide polymorphism (SNP) m.1555A>G affecting the mitochondrial gene MT-RNR1 in human buccal cells. The Genedrive® platform is a portable, rapid thermocycling, point of care test (POCT) instrument which we programmed to perform loop-mediated isothermal amplification (LAMP) nucleic acid amplification followed by end-point fluorescent hybridisation probe-based melt analysis detection to enable allelic discrimination.

The CE-IVD assay was designed to amplify the m.1555A>G region from a buccal swab sample suspended in a buffer solution, following reconstitution of lyophilised assay reagents contained in a sample cartridge (2-30ºC storage), and LAMP before genotyping wild type (WT) and mutant (VAR) alleles by automated discrimination of the respective allele hybridisation probe melt temperature (Tm) position (eFigure 1). Sample analysis is automated without any user interpretation, providing the user with a simple "detected" or "not detected" actionable result in 26 mins of initiating sample analysis (~30 mins from sample collection to actionable result). The IFU and instructional video is available at https://www.genedrive.com/assays/rnr1-product.php).

Each individual Genedrive MT-RNR1 ID Kit contains all the reagents and accessories required for the detection of (SNP) m.1555A>G affecting the mitochondrial gene MT-RNR1 in human buccal cells. The Genedrive MT-RNR1 ID Kit is designed for use in conjunction with the Genedrive instrument and an android smartphone device running the Genedrive Connect application (in an integrated device version for commercial roll out). The Genedrive MT-RNR1 ID Kit contains a buccal swab and sample collection tube containing lysis buffer for the collection of buccal cells.

The Genedrive assay cartridge contains all the reagents needed to perform amplification and detection, in a lyophilised format. The lyophilised reagents are reconstituted with the lysis buffer using the Minivette. The cartridge is then closed and inserted into the Genedrive instrument, where the required metadata is inputted via the Genedrive Connect Application, prior to initiating the test. The test consists of a target DNA amplification step and an end-point melt curve analysis. The data is interpreted by the instrument software which displays the result interpretation clearly on the screen.

© 2022 McDermott JH et al. *JAMA Pediatrics.*

Genedrive MT-RNR1 ID Kit should be stored between 2°C to 30°C. The expiry date for each Genedrive MT-RNR1 ID Kit is identified on the kit label. Expired kits must not be used. Once the Genedrive assay cartridge has been removed from the mylar pouch and the strip cap has been removed it is stable up to 40°C for 3 hours.

1.2 Assay Inclusivity

Using a panel of 92 genomic DNA samples (Public Health England, Ethnic Diversity panel EDP-1, Catalogue No: 07020701) representing a number of ethnic backgrounds including: African, Oriental, Australian Aborigine, Thai, Italian, French, South American Indian, Japanese and Ashkenazi Jewish, the MT-RNR1 assay was shown to have 100% inclusivity across all ethnicities.

1.3 Analytical sensitivity

The analytical sensitivity, or Limit of detection (LoD), is defined as the lowest concentration which >95% of the tested samples generate a positive result and was determined to be 16 cells.

Whilst the clinical significance of reported heteroplasmy is unclear, the Genedrive MT-RNR1 ID Kit can detect the variant allele (m.1555G) at a level of 10% in a background of non-variant (m.1555A), with 100% detection rate.

1.4 Interfering substances

The effect of potentially interfering substances on the Genedrive MT-RNR1 ID Kit was evaluated using 18 potentially interfering substances spiked into adult and neonatal/infant buccal samples of non-variant (1555A) at a level predicted to be above the concentration likely to be found in a buccal sample. The substances tested are shown in eTable 1.

		m.1555A Results			
		Adult		Neonate	
Substance	Concentration	Expected	Actual	Expected	Actual
Lysozyme	54.1 mg/L	8	8	8	8
Lactoferrin	29 mg/L	8	8	8	8
Glucose	14.7 μmol/L	8	8	8	8
Urea	29.3 mmol/L	8	8	8	8
Ammonia	329 mmol/L	8	8	8	8
Mucin	2270 mg/L	8	8	8	8
Albumin	149.2 mg/L	8	8	8	8
Lactate	0.79 mmol/L	8	8	8	8
α amylase	897 U	8	8	8	8
Amino acids	1560 μmol/L	8	8	8	8
IgA	156.5 mg/L	8	8	8	8

© 2022 McDermott JH et al. *JAMA Pediatrics.*

**Whole blood**	0.90%	4	4	4	4
**Amniotic fluid**	3.60%	*na*	*na*	4	4
**Meconium**	10 mg/mL	*na*	*na*	4	4
**Breast milk**	1.80%	*na*	*na*	4	4
**Orange juice**	0.90%	4	4	*na*	*na*
**Apple juice**	3.60%	4	4	*na*	*na*
**Natural yoghurt**	10 mg/mL	4	4	*na*	*na*

eTable 1. Interfering substances

1.5 Exclusivity (Cross reactivity)

The effect of potential cross reactive commensal organisms was evaluated on the Genedrive MT-RNR1 ID Kit using gut, oral, skin and vaginal microbiome genomic DNA pools (ATCC MSA-1006, MSA-1004, MSA-1005, MSA-1007 respectively). 100% exclusivity was observed with 10^5 genome copies/strain of all the organisms listed in eTable 2. False positive reactivity was observed with 10^6 genome copies/strain using the gut microbiome sample pool in 3/7 replicate tests. However, since these false positive peaks were observed in the WT position there is no impact on the result of the MT-RNR1 test. No interference of expected test result was observed when challenging the assay at 3X LoD with 10^6 genome copies/strain of each pool.

		**Cross Reactivity**		**Interference**
**Sample Pool**	**Strain**	**10^5 genome copies per strain per reaction**	**10^6 genome copies per strain per reaction**	**10^6 genome copies per strain per reaction**
**Gut Microbiome**	*Bacteriodes fragilis* (ATCC 25285D-5)	No reactivity	Reactivity in WT position (3/7 replicates)	No Interference
	Bacteriodes vulgatus (ATCC 8482D-5)			
	Bifidobacterium adolescentis (ATCC 15703D-5)			
	Clostridioides difficile (ATCC 9689D-5)			
	Enterococcus faecalis (ATCC 700802D-5)			
	Lactobacillus plantarum (ATCC BAA-793D-5)			
	Enterobacter cloacae (ATCC 13047D-5)			
	Escherichia coli (ATCC 700926D-5)			
	*Helicobacter pylori (*ATCC 700392D-5)			
	Salmonella enterica subsp. enterica (ATCC9150D-5)			
	Yerinia enterocolitica (ATCC 27729D-5)			

© 2022 McDermott JH et al. *JAMA Pediatrics.*

	Fusobacterium nucleatum subsp. nucleatum (ATCC 25586D-5)			
**Oral Microbiome**	*Actinomyces odontolyticus* (ATCC 17982D-5)	No reactivity	No reactivity	No Interference
	Prevotella melaninogenica (ATCC 25845D-5)			
	Fusobacterium nucleatum subsp. nucleatum (ATCC 25586D-5)			
	Streptococcus mitis (ATCC 49456D-5)			
	Veillonella parvula (ATCC 17745D-5)			
	Haemophilus parainfluenzae (ATCC 33392D-5)			
**Vaginal Microbiome**	*Gardnerella vaginalis (*ATCC 14019D-5)	No reactivity	No reactivity	No Interference
	Lactobacillus gasseri (ATCC 33323D-5)			
	Mycoplasma hominis (ATCC 23114D-5)			
	Prevotella bivia (ATCC 29303D-5)			
	Streptococcus agalactiae (ATCC BAA-611D-5)			
	Lactobacillus jensenii (ATCC 25258D-5)			
**Skin Microbiome**	*Acinetobacter johnsonii* (ATCC 17909D-5)	No reactivity	No reactivity	No Interference
	Corynebacterium striatumi (ATCC 6940D-5)			
	Micrococcus luteus (ATCC 4698D-5)			
	Cutibacterium acnes (ATCC 11828D-5)			
	Staphylococcus epidermidis (ATCC 12228D-5)			
	Streptococcus mitis (ATCC 49456D-5)			

eTable 2. Cross Reactivity

1.6 Diagnostic sensitivity and specificity

Prior to the implementation study, a case-control clinical performance study was conducted using prospectively collected and banked neonatal/infant and adult buccal samples. Due to the low prevalence of the m.1555A>G variant, a case-control design was chosen to allow for sufficient recruitment of cases to assess sensitivity of the test.

A total of 306 samples (sequenced) were available, with approximately one third of these representing neonatal buccal samples. Samples were prospectively collected. All 1555G variant samples were from adults. 10 samples returned "Test Failed" results on first test (3.27% test fail rate), which on retest reduced to 2 "Test Failed" results

© 2022 McDermott JH et al. *JAMA Pediatrics.*

(0.65% test fail rate), resulting in 304 samples from 159 donors included in diagnostic performance assessment (eTable 3).

- Group 1 CONTROLS – Individuals who are already known to not carry the m. 1555A>G genetic variant, as previously tested on a blood sample through the normal clinical laboratory process. This group comprised seventy-four individual participants and 138 individual specimens.
- Group 2 CASES – Individuals who are already known to carry the m.1555A>G genetic variant as this will have previously been tested on a blood sample through the normal clinical laboratory process. These individuals collected buccal cells themselves remotely following instructions supplied. This group comprised thirty-two individual participants and sixty-two individual specimens.
- Group 3 CONTROLS – Children on the neonatal intensive care unit (NICU). These samples will be used to ensure there are no factors specific to neonatal swab sampling that would impair the assay. This group comprised fifty-five individual participants and 110 individual specimens.

In this case-control, the Genedrive MT-RNR1 ID Kit was validated for use in adult and neonatal populations to detect the MT.1555A>G SNP associated with aminoglycoside induced hearing loss. The sensitivity of the test is determined to be 100 % (95 % CI 93.9 to 100.0) and the specificity is determined to be 100 % (98.5 to 100.0), using sanger sequencing as the reference method.

		**Cohort following Excluded samples**	
**Group**	**Details**	**Number of Participants**	**Number of specimens**
**1**	Adults with the MT-RNR1 A variant	74	138
**2**	Adults with the MT-RNR1 G variant	32	60
**3**	Neonates with the MT-RNR1 A variant	53	106
		159	304

eTable 3. Pre-clinical Assay Validation Case Control Study Participants

© 2022 McDermott JH et al. *JAMA Pediatrics.*

2. Study Committee Membership
2.1 Trial Management Committee: Monthly Meetings

Name	Institution	Role
Professor William Newman (PI)	Manchester Centre for Genomic Medicine, Manchester	Consultant Clinical Geneticist & Professor of Translational Genomics
Dr John McDermott	Manchester Centre for Genomic Medicine, Manchester	Clinical Research Fellow
Professor Richard Body	Emergency Department, Manchester Royal Infirmary, Manchester University NHS Foundation Trust	Consultant Emergency Medicine
Professor Iain Bruce	Paediatric ENT Department, Royal Manchester Children's Hospital	Consultant Paediatric Otolaryngologist & Professor of Paediatric Otolaryngology
Mr Peter Roberts	Market Access & Reimbursement Solutions Ltd	Value Analyst
Mr Duncan Stoddard	DS Analytics, London, UK	Data Analyst
Ms Rachel Corry	N/A	Parent Representative
Ms Julia Garlick	N/A	Parent Representative
Dr Ajit Mahaveer	Neonatal Intensive Care Unit, St Mary's Hospital, Manchester	Lead Consultant Neonatologist (St Mary's)
Dr Nicola Booth	Neonatal Intensive Care Unit, St Mary's Hospital, Manchester	Lead NICU Research Nurse (St Mary's)
Professor Mark Turner	Neonatal Intensive Care Unit, Liverpool Women's Hospital	Lead Consultant Neonatologist (Liverpool Women's)
Dr Gino Miele	Genedrive Diagnostics Ltd, Manchester, UK	R&D Director
Dr Sarah Barnett	Genedrive Diagnostics Ltd, Manchester, UK	Senior Product Manager
Dr Rachel Mahood	Manchester Centre for Genomic Medicine, Manchester	Project Manager
Dr Karen Tricker	Manchester Centre for Genomic Medicine, Manchester	Project Manager
Dr Rhona Macleod	Manchester Centre for Genomic Medicine, Manchester	PI for Staff Interview Study
Dr Fiona Ulph	Division of Psychology & Mental Health, University of Manchester	PI for Parent Interview Study
Mr Paul Wilson	Alliance Manchester Business School, University of Manchester	Implementation Scientist

eTable 4. Trial Management Committee: Monthly Meetings

2.2 Steering Committee: 6 Monthly Meetings

Name	Institution	Role
Professor Sir Munir Pirmohamed (Chair)	University of Liverpool	Professor of Pharmacology & Therapeutics
Professor Andrew Ewer	University of Birmingham	Professor of Neonatal Medicine
Dr Jonathan Sandoe	University of Leeds	Associate Professor of Microbiology and Honorary Consultant Microbiologist
Dr Jane Theaker	Kinomica Ltd	Chief Executive Officer
Professor Dyfrig Hughes	Bangor University	Professor in Pharmacoeconomics
Dr Lynne Webster	Manchester University NHS Foundation Trust	Head of Research & Development Office

eTable 5. Steering Committee: 6 Monthly Meetings

© 2022 McDermott JH et al. *JAMA Pediatrics.*

2.3 Stakeholders Committee

Name	Institution	Role
Vicki Kirwin (Chair)	National Deaf Children's Society	Development Manager
Professor Kevin Munro	University of Manchester	Professor of Audiology
Ms Christine Ashworth	Newborn Services MFT	Directorate Manager
Ms Rachel Ward	Manchester University NHS Foundation Trust	Paediatric Audiologist
Ms Lucy Culshaw	Bliss (National Charity for babies born premature or sick)	Charity representative
Ms Sonali Sanghvi	Pharmacy Advisor	Genomics Unit at NHS England
Ms Lisa Juniper	N/A	Parent Representative
Ms Laura Hawcroft	N/A	Parent Representative
Mr Paul Wilson	Alliance Manchester Business School, University of Manchester	Implementation Scientist

eTable 6. Stakeholders Committee

3. Inclusion & Exclusion Criteria

3.1 Inclusion criteria

All babies admitted to NICU across the two participating sites, commencing from the trial start date. This includes babies admitted directly from delivery suite, post-natal wards, midwifery led units or transferred from another neonatal unit. At the LWH site, babies who were screened for infection on the NICU but then transferred to external wards (not formally admitted to NICU) were also included. It is a clinical decision whether the babies are being screened for infection as part of their assessment, reflecting the real-world, pragmatic, nature of the trial. This also reflected the variation in admission procedures between the two trial sites where pathways differ due to local practices.

3.2 Exclusion Criteria

Babies requiring antibiotics immediately, as determined by the admitting clinician, with already established intravenous (IV) access. These exclusion criteria recognize the potential urgency of any decision to deliver antibiotics. If IV access has not been achieved, then this may provide time to run the assay. We expect that only a small proportion of admissions would be excluded based on these criteria.

4. MT-RNR1 Point of Care System Investigations

During the implementation phase there was "Test fail" rate of 17.4%, where genotyping failed, and five false positive events. The study design allowed genotyping data to be collected from the PALOH implementation trial to inform assay modification to improve the final system performance.

4.1 Failed Genotyping Investigations

© 2022 McDermott JH et al. *JAMA Pediatrics.*

The samples which had failed genotyping ("Test Fail") during the PALOH trial were retrospectively reviewed. There was no association between failed genotyping and participant age, length of time between swab and testing, system, system user, assay batch or the time of day the test was performed. Subsequently, the melt peak data for those samples which had failed genotyping were retrospectively reviewed. A consistent feature of those samples which failed genotyping was a reduced signal intensity below acceptance threshold, meaning the peaks were not being identified by the system software and genotyping would fail. This appeared to be a root-cause of the increased "Test Fail" rate. Rather than lowering the threshold for a peak to be considered detected, which could reduce assay sensitivity, the assay buffer formulation was modified to improve analytical sensitivity, and subsequently lowered the assay LoD from 16 cells to 0.4 cells.

This version 2.0 (V2.0) chemistry was then tested on 35 patient samples which had all previously failed to generate a genotype (i.e. Test Fail) during the PALOH implementation trial. All samples irrevocably anonymised prior to repeated testing. The peak heights and outcome from each genotyping attempt was monitored. All samples were tested in a controlled laboratory setting and in the clinical setting, where the PALOH trial was performed.

Repeated testing of the 35 previously failed samples showed a significant improvement in the performance of the system, both in the laboratory and clinical settings. The average peak height was significantly increased using V2.0 of the MT-RNR1 assay (eFigure 2) which translated into a decreased Test Fail rate. In the laboratory setting, all of the previously failed samples were successfully genotyped (failure rate 0.0%) and in the clinical setting only 2 of 35 samples failed genotyping (failure rate 5.7%)

4.2 False Positive Event Investigations

Repeat testing of samples leading to false positive events ruled out sample contamination. False positive results were associated with irregular melting profile traces, with spikes and dips introduced into the fluorescent melt profile. Fluorescence spikes resulted in artefactual "peaks" passing subsequent quality metrics and fluorescent dips or dips resulted in possibility of masking true melt peaks, and therefore also potentially contributing to "test fail" results observed. These were determined to arise from incomplete assay cartridge insertion, resulted in vibrational derived artefacts and light ingress to the optical reader system during assay melt phase. The cartridge and cartridge inlet design were improved, with physical clip and auditory feedback to the user to ensure that these were inserted correctly. Following verification and confirmation of new design improvements, these were validated in the laboratory and intended user setting as above. Instructions for use (IFU) and a user video, demonstrating ideal use of the system including cartridge insertion, are available at https://www.genedrive.com/assays/rnr1-product.php.

© 2022 McDermott JH et al. *JAMA Pediatrics.*

4.3 Investigation Conclusion

Assay and instrument/assay cartridge design improvements have led to substantial improvement in assay analytical sensitivity, thereby addressing the "test fail" rate, and ensuring correct and complete cartridge assembly and insertion, thereby addressing potential false positive events derived from vibrational or light ingress related fluorescence artefacts. These post-implementation study identified design improvements have led through to commercially available CE marked product.

5. Supplementary (Online-Only) eFigures

5.1 eFigure 1

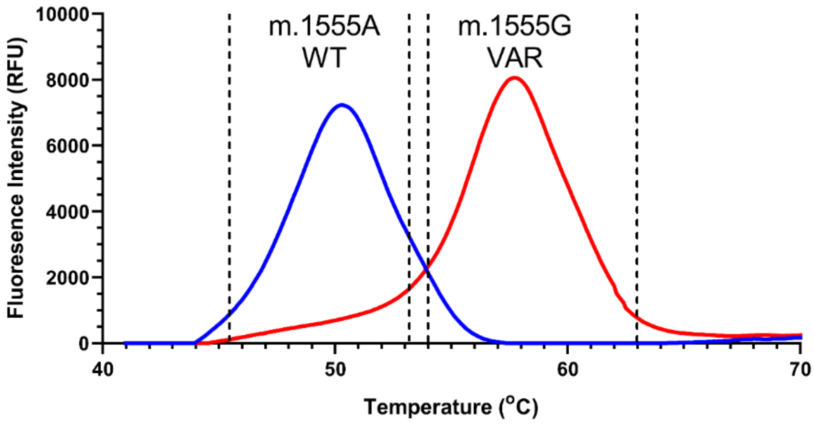

eFigure 1 Melt Curve Analysis. Differential peak heights between the m.1555A (WT) and the m.1555A>G (VAR) alleles, allowing reliable discrimination during genotyping.

5.2 eFigure 2

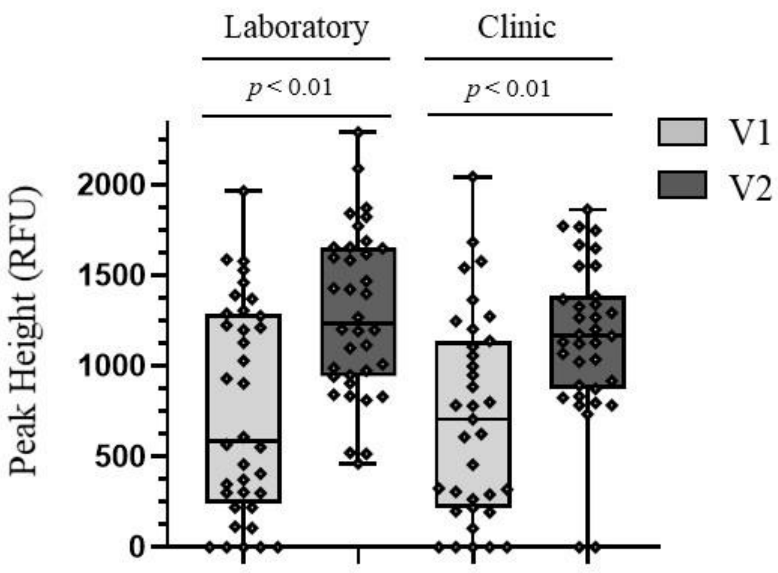

eFigure 2 Peak Height Assessment. Reformulation of the buffer chemistry for version 2.0 of the assay significantly increased the average peak height compared with version 1.0 of the assay. RFU= relative fluorescence units

© 2022 McDermott JH et al. *JAMA Pediatrics.*
