# Supplementary material for: Rapid Point-of-Care Genotyping to Avoid Aminoglycoside-Induced Ototoxicity in Neonatal Intensive Care
Source: JAMA Pediatr. 2022 Mar 21;176(5):486–92. doi: 10.1001/jamapediatrics.2022.0187 (PMC8938898; doi:10.1001/jamapediatrics.2022.0187)
Supplement: Supplement 2. — Nonauthor Collaborators. The PALOH Study Team members [file jamapediatr-e220187-s002.pdf]

Supplemental Online Content: Nonauthor Collaborators
*First name, last name, and suffix (if applicable) are required and will appear in PubMed.

***Group Name(s): The PALOH Study Team**

***First Name and Middle Initial(s)**	***Last Name**	***Suffix (eg, Jr, III)**	Academic Degrees	Institution	Location (city, state/province, country)	Role or Contribution, eg, chair, principal investigator	Group (if more than 1 Group listed in the byline) and/or Subgroup (eg, Steering Committee)
Imelda	Mayor		RN	St Mary's Hospital	Manchester, UK	Research Nurse	
Clare	Jennings		RN	St Mary's Hospital	Manchester, UK	Research Nurse	
Karen	Dockery			St Mary's Hospital	Manchester, UK	Research Coordinator	
Jenna	Hill		RN	St Mary's Hospital	Manchester, UK	Research Nurse	
Joanne	Windrow		RN	Liverpool Womens Hospital	Liverpool, UK	Research Nurse	
Patrick	McGowan		RN	Liverpool Womens Hospital	Liverpool, UK	Research Nurse	
Amy	Ingham			St Mary's Hospital	Manchester, UK	Research Coordinator	
Sarah	Rushton			St Mary's Hospital	Manchester, UK	Research Coordinator	
Poly	Kirkilli			St Mary's Hospital	Manchester, UK	Research	
Suzanne	Parsons			Manchester University NHS	Manchester, UK	Public and Patient	
Ruth	Gottstein		FRCPCH	St Mary's Hospital	Manchester, UK	Consultant	
Ngozi	Edi-Osagie		FRCPCH	St Mary's Hospital	Manchester, UK	Consultant	
Christine	Ashworth		RN	St Mary's Hospital	Manchester, UK	Director - Newborn	
Maxine	Brandall		RN	St Mary's Hospital	Manchester, UK	NICU Sister	
Kath	Eaton		RN	St Mary's Hospital	Manchester, UK	Lead nurse -	
